# Adaptive Shannon Entropy-Driven Chaotic Grey Wolf Optimization (ASE-CGWO) for Combinatorial Optimization Problems

**DOI:** 10.3390/biomimetics11100689

**Published:** 2026-09-23

**Authors:** Simi Kalathodi, Sarada Jayan, Bilal Alatas, Mehmet Das, Merve Celebi, Ebru Akpinar

**Affiliations:** 1 Department of Mathematics, Amrita School of Engineering, Amrita Vishwa Vidyapeetham, Bengaluru 560035, Karnataka, India; bl.sc.d.mat16008@bl.students.amrita.edu (S.K.); j_sarada@blr.amrita.edu (S.J.); 2 Department of Software Engineering, Firat University, 23119 Elazig, Türkiye; balatas@firat.edu.tr; 3 Department of Mechanical Engineering, Firat University, 23119 Elazig, Türkiye; ebruakpinar@firat.edu.tr; 4 Department of Computer Technologies, Antakya Vocational School, Hatay Mustafa Kemal University, 31060 Antakya, Türkiye; merveorakci@mku.edu.tr

**Keywords:** combinatorial optimization, grey wolf optimization (GWO), chaos, entropy-based adaptive control, metaheuristic optimization, elite memory strategy, neighborhood refinement, benchmark optimization problems

## Abstract

Combinatorial optimization problems include many NP-hard problems such as assignment, scheduling, and routing, for which conventional approaches to solving become computationally infeasible for large instances, thereby necessitating the use of metaheuristics. This study introduces an algorithm, Adaptive Shannon Entropy-Driven Chaotic Grey Wolf Optimization (ASE-CGWO), for combinatorial optimization problems. The proposed approach adapts the level of chaos using an entropy-based diversity measure to ensure an efficient trade-off between exploration and exploitation. It also uses an entropy-adapted hunting parameter that couples exploration breadth directly to the pack’s information content. An Elite Memory Archive and swap, along with insertion and flip operators to enhance neighborhood searches, are also incorporated to maintain high-quality solutions. ASE-CGWO is benchmarked against Standard GWO, Chaotic GWO (fixed r = 3.9 logistic map), Entropy-Adaptive GWO (entropy adapts “*a*” only), Genetic Algorithm (GA), Particle Swarm Optimization (PSO), and Ant Colony Optimization (ACO) on three NP-hard combinatorial optimization problems: the Quadratic Assignment Problem instance chr15a, the Fisher–Thompson 10 × 10 Job Shop Scheduling Problem (FT10) and the 20-city Traveling Salesman Problem (TSP20) over 20 independent runs each. ASE-CGWO achieves the best mean assignment cost on chr15a (9921.6 ± 147.3 in native QAPLIB units), with its best run reaching the known optimum of 9896, and obtains the second-best mean makespan on FT10 (1182.05 ± 3.32). The adaptive and self-regulating characteristics of ASE-CGWO also suggest potential for future applications in complex engineering optimization problems, including the design and operation of solar energy systems.

## 1. Introduction

The combinatorial optimization problems appear in a variety of practical applications, such as scheduling problems, routing problems, allocation problems, Knapsack problems, feature selection problems, etc. [1,2]. In these problems, it is required to find the optimal solution among many combinations under some constraints [3]. Because of the exponential increase in the problem space size, the exact optimization becomes very complex as the problem size increases. Metaheuristics are now widely used owing to the capacity of finding near-optimal solutions efficiently [4]. The currently available metaheuristics include PSO, GA, ACO Differential Evolution (DE) and GWO, among others, all showing potential in the optimization process [5]. However, most of these metaheuristics use static parameters for control purposes and pre-defined searching techniques that limit their flexibility in adapting to different problem landscapes [6]. Additionally, traditional optimization techniques tend to experience premature convergence and lack balanced exploration–exploitation trade-offs.

Chaotic optimization algorithms have been proposed to increase the diversity of search processes and optimize the abilities of global search. Ergodic and random behaviors in chaotic maps have the ability to avoid the problem of being trapped in local optimum during optimization [7,8,9]. But many methods that use chaotic behavior in optimization use chaotic systems in a simple way, without paying attention to the dynamical condition of the population. Furthermore, there is no mechanism to control the intensity of chaotic behavior, and, thus, they cannot be applied in many cases. New research has revealed the effectiveness of chaotic map dynamics in increasing the adaptive behavior and self-organization of optimization methods. The application of chaotic map dynamics may generate nonlinear and even chaotic behaviors, which may result in better performance during the exploration phase of the algorithm. But the use of chaos in combination with entropy-based adaptive controllers has not received much attention. Entropy is an appropriate tool for evaluating the diversity of the population, and it can effectively control the level of chaos [10].

Grey Wolf Optimization (GWO) proposed by Mirjalili et al. in 2014 [11] is inspired by the leadership hierarchy and cooperative hunting behavior of grey wolves (Canis lupus). Due to its simplicity, computational efficiency, and competitive optimization performance, GWO has become one of the most widely adopted population-based metaheuristic algorithms and has been successfully applied to numerous continuous, discrete, multi-objective, and engineering optimization problems. The population is organized into four tiers for GWO, where the three fittest solutions (α, β, δ) collectively guide the remaining search agents toward promising regions of the search space [11].

The hunting process includes three sequential stages: encircling the prey, cooperative hunting, and attacking. These stages are governed by a linearly decaying control parameter that progressively transitions the search from broad exploration to refined exploitation at later iterations. A notable strength of GWO is its mathematical simplicity: it requires minimal control parameters and no complex genetic operators such as crossover or mutation, making it straightforward to implement and computationally efficient. These properties have driven its widespread adoption across diverse domains, including feature selection, scheduling, power systems, image processing, and structural optimization, where it has consistently demonstrated competitive or superior performance relative to established algorithms such as PSO, DE, GSA, and evolutionary strategies [12,13]. Despite its effectiveness, the standard GWO exhibits two inherent shortcomings that can limit its optimization performance. The first is its tendency toward premature convergence. Since the control parameter ‘***a***’ decreases according to a predetermined linear schedule, the balance between exploration and exploitation is fixed throughout the optimization process rather than adapting to the current state of the population. As a result, the search may lose diversity too quickly and become trapped in local optima, particularly when solving complex multimodal problems. The second limitation is the progressive loss of search information. During each iteration, new candidate solutions are generated primarily under the guidance of the three best wolves, while information contained in the remaining population is not preserved. Consequently, potentially useful search experiences are discarded, which can reduce solution diversity and weaken the algorithm’s ability to discover alternative promising regions of the search space.

This study addresses both limitations by introducing ASE-CGWO, which replaces the linear ‘***a***’ schedule with an entropy-driven chaos mechanism, adds an elite archive for knowledge preservation, and embeds a tri-operator neighborhood search for local intensification. The result is a self-adapting framework that responds to the wolf pack’s information state rather than following a pre-specified schedule.

### Key Contributions

A key contribution is the creation of the first entropy-controlled chaotic GWO for combinatorial optimization with an adaptive hunting parameter, memory archive and a tri-operated neighborhood search.

The relative performance analysis of ASE-CGWO was carried out against several well-established metaheuristic algorithms using solution quality, convergence behavior, computational efficiency, and statistical significance tests.

The complete evaluation of the proposed ASE-CGWO algorithm on standard combinatorial optimization benchmark problems was performed to assess its robustness, effectiveness and the convergence characteristics.

The remainder of this paper is structured as follows. Section 2 presents a comprehensive review of the relevant literature; Section 3 provides the background of this study, including the explanation of the three benchmark problems used for testing the results; and Section 4 introduces the proposed algorithm, ASE-CGWO, in detail. Section 5 outlines the experimental setup, and Section 6 presents and analyzes the experimental results with a detailed discussion. Finally, this study is concluded in Section 7.

## 2. Literature Review

Mirjalili et al. [11] introduced GWO by formalizing the grey wolf social hierarchy into encircling equations driven by coefficient vectors A and C derived from the linearly decaying ‘*a*’. Emary et al. [14] adapted GWO for binary feature selection using V-shaped and S-shaped transfer functions. Kaveh and Zakian [15] applied GWO to combinatorial structural optimisation using permutation-based position updates. Li et al. [16] enhanced the Grey Wolf Optimization algorithm by integrating lens opposition-based learning with an adaptive nonlinear convergence factor to improve population diversity and balance exploration and exploitation, while Qiu et al. [17] proposed an improved GWO that combines adaptive search weighting, Cauchy mutation, and an elite-guided update strategy to strengthen global exploration and local exploitation. Both these demonstrated improved convergence accuracy and higher solution quality than the standard GWO and several recent metaheuristic algorithms on functional and engineering optimization problems. The discrete position update—blending swap sequences toward the three leaders—used in this study follows the swap-sequence formulation of Clerc [18] for PSO, adapted to GWO’s three-leader structure.

Several studies have explored hybrid variants of GWO by integrating it with complementary metaheuristics, including GA, Differential Evolution, PSO, Simulated Annealing, Artificial Bee Colony, Cuckoo Search, Whale Optimization, and ACO, with the general objective of coupling GWO’s broad exploratory capability with the local search strengths of the partner algorithm [19,20].

Kohli and Arora in [21] introduced CGWO by substituting the logistic map for the linearly decaying a, reporting improvements on continuous benchmark functions. Wang et al. used tent-map chaos for binary GWO feature selection [22]. However, all prior chaotic GWO variants fix the map parameter r, causing the chaos to be invariant to the population’s state. ASE-CGWO is the first work to make r a continuous function of the swarm’s Shannon entropy diversity, creating a feedback-controlled chaos mechanism.

The use of Shannon entropy as a measure of population diversity was first explored by Morrison and De Jong, providing a quantitative means of assessing the search state during optimization [23]. Later, Hu et al. employed entropy-based adaptive parameter adjustment in PSO to improve task scheduling performance [24]. For GWO, prior entropy-adaptive variants adapt only ‘*a*’ or the coefficient vectors, without coupling entropy to an underlying chaotic map. The EGWO baseline in this study isolates this contribution: using entropy to adapt ‘*a*’ without chaos yields substantially worse results on most benchmarks, confirming the necessity of the chaos–entropy coupling.

Elite preservation has been widely adopted in population-based optimization to retain high-quality solutions and improve convergence. While Genetic Algorithms employ simple elitism and MOPSO [25] maintains Pareto archives, CLPSO [26] enables particles to learn from multiple historical exemplars. Motivated by these approaches, the proposed Elite Memory Archive (EMA) preserves multiple elite solutions and selects search guides using a rank-based strategy. Similarly, neighborhood search has been shown to enhance solution quality, with operators such as 2-opt, Or-opt insertion, and other local search techniques improving convergence in combinatorial optimization problems [27,28]. Accordingly, the proposed method integrates Swap, Insertion, and Flip operators to strengthen local search during optimization.

The combinatorial optimization literature has also been extended to non-classical problem domains. Liu and Gao [29] proposed an improved puzzle optimization algorithm with multi-strategy integration, demonstrating the applicability of adaptive metaheuristic design principles to puzzle-type combinatorial structures beyond conventional scheduling and assignment benchmarks. Scarrica [30] studied the enumerative combinatorics of row-convex polyominoes via partition-based generating functions, highlighting the richness and diversity of combinatorial structures that motivate the development of general-purpose, adaptive optimization frameworks such as the one proposed in this study. The main algorithmic features of the proposed ASE-CGWO and the benchmark GWO variants are summarized in Table 1.

## 3. Preliminaries

### 3.1. Grey Wolf Optimization

The hunting process in the standard GWO uses the parameter

(1) $$a\left(t\right)=2-\frac{2t}{T}$$

where *t* is the current iteration and *T* is the maximum number of iterations.

This is a linearly decaying parameter and is used to compute the coefficient vectors *A* and *C*, as in Equation (2), that randomize encircling distances:

*A* = 2*ar*_1_, *C* = 2*r*_2_,  where *r*_1_, *r*_2_ ~ *U*(0,1) (2)

Every grey wolf estimates its distance from the prey before updating its position using the equation:

D = ∣C · X*_p_*(*t*) − X(*t*)∣ (3)

where D is the distance vector between the prey and the wolf, X_p_(t) is the prey position and *X*(t) is the current wolf position. But since the prey position X_p_(t) is unknown, the position of the three leaders *α*, *β* and *δ*, namely, X_α_(t), X_β_(t) and X_δ_(t), respectively, is considered as in Equation (4)

*D_α_* =|*C*_1_*X_α_*(*t*) − *X*(*t*)|, *D_β_* = |*C*_2_*X_β_*(*t*) − *X*(*t*)|, *D_δ_* = |*C*_3_*X_δ_*(*t*) − *X*(*t*)| (4)

The updated position of each wolf is then obtained by combining the guidance provided by the three highest-ranked wolves, *α*, *β* and *δ*, as:

(5) $$X\left(t+1\right)=\frac{X_{1}+X_{2}+X_{3}}{3}$$

where *X*_1_, *X*_2_ and *X*_3_ are, respectively, the candidate positions generated with respect to *α*, *β* and *δ* foxes obtained using equations:

*X*_1_ = X*_α_*(*t*) − A_1_ · D_α_, *X*_2_ = X*_β_*(*t*) − A_2_ · D_β_, *X*_3_ = X*_δ_*(*t*) − A_3_ · D_δ_ (6)

In the discrete version of GWO, wolf positions are represented as permutations π_i_ rather than continuous vectors. Position updates are implemented by applying swap-based transformations that progressively modify the current solution toward the corresponding leader permutation. The detailed procedure is given as an algorithm in Section 4.4.

### 3.2. Shannon Entropy

The Shannon entropy is a mathematical measure of uncertainty widely used to quantify the diversity of a population. It is computed as:

*H*(*t*) = −*Σ p_i_*(*t*)*ln*(*p_i_*(*t*)) (7)

Only probabilities greater than zero are included in the summation in order to avoid the undefined Logarithm of zero. For a population of size N, if the fitness values in an iteration are given

*F*(*t*) = {*f*_1_(*t*), *f*_2_(*t*), …, *f_N_*(*t*)},

the probability *p_i_* is defined as:

(8) $$p_{i}\left(t\right)=\frac{\tilde{f_{i}}\left(t\right)}{\Sigma \left(\tilde{f_{i}}\left(t\right)\right)},\mathrm{where}\tilde{f_{i}}(t)=f_{i}(t)-\min(F(t))\;$$

High entropy indicates a highly diverse population, and low entropy signifies convergence. The maximum entropy occurs when the population distribution is uniform, i.e., when

$$p_{i}=\frac{1}{N}$$

Substituting into Shannon’s equation the theoretical upper bound of population diversity can be obtained as *H_max_* = *ln*(*N*).

A normalized diversity measure independent of population size is obtained by dividing the entropy by its maximum value:

(9) $$d\left(t\right)=\frac{H\left(t\right)}{\ln\left(N\right)}$$

So that *d*(*t*)∈ [0, 1]. The interpretation of the diversity measure of a population in optimization search is explained in Table 2.

In the proposed work, the diversity index at every iteration simultaneously controls

1. The logistic-map parameter r(t).
2. The Grey Wolf hunting parameter a(t).

Scope and interpretation of fitness-based entropy: The Shannon entropy defined in Equation (7) characterizes the distribution of fitness values within the population and should not be interpreted as a direct measure of the spatial diversity of solutions in the permutation search space. Different permutations may produce similar or identical objective values, while solutions with similar fitness may differ substantially in their permutation structures. Nevertheless, fitness-based entropy provides a computationally efficient indicator of the population’s objective-value distribution, which can be used to guide the balance between exploration and exploitation in ASE-CGWO. In the proposed approach, changes in fitness diversity are used to regulate the chaos intensity and search behavior, rather than being assumed to represent the exact spatial dispersion of the population. Position-based diversity measures, such as average pairwise Kendall-tau distance, could provide complementary information about the structural diversity of candidate solutions. Incorporating such measures and investigating their influence on the adaptive search mechanism remain potential directions for future research.

### 3.3. Logistic Map

Logistic maps are the most popular type of chaotic map used in various optimization problems in the literature. It is defined as:

*x*(*t* + 1) = *r* · *x*(*t*) · (1 − *x*(*t*)) (10)

Here, *r* is the control parameter, and in most of the algorithms, it is chosen as a fixed value, either 4 or 3.9. The proposed algorithm suggests keeping *r* as a varying measure *r*(*t*) that depends on the diversity of the population in that iteration.

The logistic map demonstrates different dynamical behaviors depending on the control parameter *r* [31]. At lower values of *r*, the system approaches stable fixed points, followed by periodic oscillations and period-doubling bifurcations as *r* increases. Beyond approximately *r* = 3.57, chaotic dynamics emerge, characterised by aperiodic trajectories and sensitivity to initial conditions. Values such as *r* = 3.9 and *r* = 4, commonly adopted in previous studies, fall within this chaotic region. In ASE-CGWO, the parameter bounds are set to *r*_min_ = 2.80 and *r*_max_ = 3.95 to enable adaptive transitions between periodic and chaotic behaviours. The lower bound supports relatively controlled perturbations that help preserve promising search directions, whereas the upper bound promotes enhanced exploration through chaotic dynamics while avoiding the boundary value *r* = 4.0, which was associated with occasional orbit degeneration requiring reseeding during preliminary experiments. Accordingly, the interval [2.80, 3.95] provides a continuous range of perturbation intensities, with the parameter *r*(*t*) adjusted according to the population Shannon entropy diversity index *d*(*t*).

### 3.4. Benchmark Problems

The details of the benchmark problems used to test the performance of the proposed algorithm are explained here.

#### 3.4.1. Quadratic Assignment Problem (chr15a)

The Quadratic Assignment Problem (QAP) [32] seeks an optimal one-to-one assignment of *N* facilities to *N* locations such that the total assignment cost, which is computed from the interaction between facility flows and inter-location distances, is minimized. Let *F* = (*F_ij_*) denote the flow matrix and *D* = (*D_ij_*) the distance matrix. The objective function is expressed as

(11) $$\mathrm{Minimize}f\left(\pi \right)=\sum_{i=1}^{N}\sum_{j=1}^{N}F_{\mathrm{ij}}\cdot D_{\pi \left(i\right),\pi \left(j\right)}$$

where *π* represents a permutation assigning facilities to locations. The Chr15a benchmark from QAPLIB comprises 15 facilities and 15 locations, with each candidate solution encoded as a permutation of the integers {0, 1, …, 14}.

#### 3.4.2. Job Shop Scheduling Problem (FT10)

The Job Shop Scheduling Problem (JSSP) deals with the process of scheduling *N_J_* jobs using *N_M_* machines, such that each job is made up of a specific set of *N_M_* operations. In the Job Shop Scheduling Problem (JSSP), each operation has to be scheduled on a designated machine for a predetermined time period such that the sequence of processing of operations within each job should be maintained. The aim of the Job Shop Scheduling Problem (JSSP) is to minimize the total time for completing all the jobs. The mathematical formulation of the optimization problem is

Minimize *C_max_*

(12) $$\mathrm{where}\;C_{\max}=\underset{1\leq i\leq N_{J}}{\max}C_{i}$$

and *C_i_* represents the completion time of job *i*.

In the Job Shop Scheduling Problem, FT10 is one of the most widely used benchmark instances. It was originally introduced by Harold Fisher and Gerhard L. Thompson [33]. The FT10 benchmark problem comprises 10 jobs and 10 machines. Each job is divided into a sequence of 10 operations, with every operation assigned to a specific machine and processed according to a fixed technological order. Consequently, the problem contains 100 operations in total.

#### 3.4.3. Travelling Salesman Problem (TSP20)

Given a set of N cities with pairwise Euclidean distances *d*(*i*,*j*), the objective of TSP [34] is to find the shortest tour between the cities {1, 2, …, N}, visiting each city exactly once. The solution is encoded as a permutation X = [x_1_, x_2_, …, x_n_], where x_i_ is the visiting order. The objective is to minimize the tour length given by

(13) $$L\left(X\right)=\sum_{i=1}^{n-1}d\left(x_{i},x_{i+1}\right)+d\left(x_{i},x_{i+1}\right)$$

The last term ensures that the salesman returns to the starting city.

TSP20 represents the standard test problem, in which there are 20 cities with predetermined intercity distances. As the problem has approximately 19!/2 ≈ 6.08 × 10^16^ feasible paths, it is a proper choice for testing the stability and search capabilities of the proposed algorithm.

## 4. Proposed Algorithm: Adaptive Shannon Entropy-Driven Chaotic Grey Wolf Optimization (ASE-CGWO)

The proposed ASE-CGWO adaptively regulates the intensity of stochastic perturbation according to the population diversity. When diversity decreases, stronger perturbations are introduced to prevent premature convergence and escape local optima, whereas weaker perturbations are applied when diversity is high to preserve promising search directions and maintain an effective balance between exploration and exploitation. In the proposed algorithm, diversity of the wolf population is quantified using an entropy measure, which is then employed to adapt the logistic chaotic map and the Grey Wolf hunting parameter during the optimization process.

### 4.1. Fitness Evaluation and Diversity Index Calculation

Initially, the fitness values are evaluated for each wolf, and then the corresponding probability *p_i_* for each wolf is determined using Equation (8). Then, Equation (7) is used to evaluate the Shannon entropy of the population followed by the computation of the diversity measure *d*(*t*)∈ [0, 1] as in Equation (9).

A diversity measure (*d*(*t*)) closer to one in an iteration of ASE-CGWO indicates that all the wolves have different fitness, indicating that the population is very diverse or ‘spread out’, in which case the search requires more exploration. *d*(*t*) closer to zero indicates all wolves have almost the same fitness and that the population state is convergent or stagnating, in which case ASE-CGWO injects chaos to escape becoming trapped. If *d*(*t*) is close to 0.5, population has moderate diversity, suggesting a balanced search. Figure 1 visualizes these three cases by showing the probability distribution of the wolves in an eight-wolf population when the diversity measure is close to 1, 0.5 and 0.

### 4.2. Entropy-Gated Chaos

ASE-CGWO uses a diversity-controlled parameter *r*(*t*) instead of the fixed parameter *r* in the logistic map given in Equation (10), thus transforming it into Equation (14)

*x*(*t* + 1) = *r*(*t*) · *x*(*t*) · (1 − *x*(*t*)) (14)

where

*r*(*t*) = *r_min_* + [*r_max_* − *r_min_*][1 − *d*(*t*)], r*_min_* = 2.80 and r*_max_*= 3.95 (15)

The logistic-map parameter in ASE-CGWO is adaptively controlled using the population diversity measure (d(t)), as defined in Equation (14). Specifically, (r(t)) varies between 2.80 and 3.95, with lower diversity leading to a higher parameter value. It is important to note that this parameter variation does not represent a uniformly increasing chaotic intensity throughout the entire range. At *r_min_* = 2.80, the standard logistic map operates in a stable fixed-point regime, whereas *r_max_* = 3.95 lies within a chaotic regime. Intermediate parameter values may correspond to periodic or chaotic dynamics, depending on the specific value of *r*(*t*). The adaptive mechanism, therefore, uses changes in the dynamical behaviour of the logistic map to regulate the sequence used in the search process. When population diversity is high, the lower parameter value produces a stable sequence, limiting the variability of the resulting perturbations. As diversity decreases, the parameter increases toward the selected upper bound, which lies in a chaotic regime, introducing more irregular sequences intended to support exploration and reduce stagnation. This mechanism is a heuristic design choice, and the relationship between the generated sequence and search performance is evaluated through the experimental results rather than being assumed to follow a strictly monotonic relationship with chaotic intensity.

### 4.3. Entropy-Adaptive Hunting Parameter

In standard GWO, the hunting parameter ‘*a*’ decreases from 2 to 0 on a fixed linear schedule (Equation (1)). ASE-CGWO proposes an adaptive entropy-controlled hunting parameter for each iteration, *a*(*t*) given by:

*a*(*t*) = 2*d*(*t*) (16)

and the normalized parameter:

*a_frac_*(*t*) = *d*(*t*) (17)

The normalized fraction *a_frac_*(*t*) = *d*(*t*) directly controls how far each wolf moves toward the leaders in one iteration. When the diversity is greater, a wolf jumps far, exploring new territory (search spaces), and with low diversity, wolves search precisely, exploiting near leaders. Figure 2 depicts how the parameters *r*(*t*) and *a*(*t*) vary with the diversity measure *d*(*t*).

### 4.4. Position Update Using Swap Sequences

In the original Grey Wolf Optimization, wolves update their positions using vector arithmetic in a continuous search space. However, such arithmetic operations cannot be directly applied to combinatorial optimization problems because a solution is represented as a permutation *π_i_* rather than a continuous vector. Therefore, the continuous position update (the velocity update) in standard GWO is replaced by a swap sequence, which is a list of pair swaps that transforms one permutation into another. One sequence is computed for each of the three leaders (α, β, δ). Each sequence contains the minimum number of swaps needed to transform *π_i_* into the leader’s permutation. Each swap from each leader’s sequence is included in the merged list (*S_merged_*) with probability proportional to its leader’s weight. With equal weights (1/3 each), each swap is included with probability 1/3. The number of swaps (*n_apply_*) to be applied is controlled by *a_frac_* from Equation (17) as:

*n_apply_* = *max* (1,⌊*a_frac_*_(*t*)_· |*S_merged_*|⌋) (18)

where |*S_merged_*| is the number of elements in *S_merged_* and ⌊1 : *n_apply_*⌋ is the floor function.

The position update would be:

(19) $$\pi_{i}^{t+1}=\mathrm{ApplySwaps}\left(\pi_{i},\;S_{\mathrm{merged}}\left[1:n_{\mathrm{apply}}\right]\right)$$

where ApplySwaps (·) sequentially applies the first *n*_apply_ swap operations in the merged swap sequence to the current position *π_i_* to obtain the updated permutation. For combinatorial problems with binary representations (like Knapsack, feature selection), which are not considered in this study, there is no permutation to swap. Instead, a majority-vote rule is used across the three leaders. Each bit in the new position is set to 1 if at least 2 of the 3 leaders have a 1 at that position, and 0 otherwise.

An additional random kick mechanism is activated, when the chaotic parameter *x*(*t*) exceeds the threshold of 0.65. When this condition is satisfied, the algorithm attempts a kick with probability *P*_kick_ = 0.35*x*(*t*). If the kick is triggered, a single randomly selected Swap or Flip operation is applied to the current wolf to introduce additional diversity. Since *x*(*t*) is generated by the chaotic logistic map, the kick mechanism is governed by the chaotic state rather than a fixed iteration schedule. Consequently, the perturbations occur at irregular, non-periodic intervals, helping the search avoid repetitive behavior and reducing the likelihood of systematic search bias or premature convergence. The discrete position update procedure using swap sequences is summarized in Algorithm 1.
**Algorithm 1.** Discrete position update via swap sequences1Input: current permutation π_i_, leaders (πα, πβ, πδ), a_frac = d(t)2S_α ← MinSwapSeq(π_i_ → πα); S_β ← MinSwapSeq(π_i_ → πβ); S_δ ← MinSwapSeq(π_i_ → πδ)3S_merged ← []4for each swap s in S_α: include s in S_merged with probability 1/35for each swap s in S_β: include s in S_merged with probability 1/36for each swap s in S_δ: include s in S_merged with probability 1/37n_apply ← max(1, floor(a_frac · |S_merged|))8π_i_(t+1) ← ApplySwaps(π_i_, S_merged [1 : n_apply])9return π_i_(t+1)

### 4.5. Neighborhood Refinement and Leader Update

In the proposed algorithm, the neighborhood update strategy operates in two modes. Every fifth iteration (t = 5, 10, 15, …), an intensive local search is performed on the current alpha wolf using 24 neighborhood probes to further refine the best solution. If a better solution is found, the alpha wolf is immediately updated. During all other iterations, a lighter local search consisting of 12 neighborhood probes is applied to the current wolf. The wolf’s position is updated only when an improved solution is obtained. In both cases, the neighborhood search employs the Swap, Insert, and Flip operators and follows a first-improvement strategy, whereby the search terminates as soon as a better neighboring solution is identified. This approach avoids unnecessary evaluations while balancing computational efficiency with solution quality. The Swap operator exchanges the elements at two randomly selected positions, enabling large structural changes in the solution. The Insert operator removes an element from one position and reinserts it at another, providing insertion-based refinements. Permutation-based solutions through local refinement are effectively improved by the Flip (2-opt) operator that reverses the subsequence between two randomly selected positions. Together, these operators maintain population diversity while improving solution quality.

Also, every 5 iterations (t = 5, 10, 15, …), an intensive neighborhood search is applied directly to the best solution α. This uses 24 probes (double the 12 used in Line 13) because α is the most promising starting point and deserves greater exploration effort.

The probe counts (12 regular/24 alpha) and frequency (every 5 iterations) were determined through a preliminary sensitivity study on chr15a using a 5-fold grid search over probe counts ∈ {6, 12, 18, 24} and frequencies ∈ {3, 5, 10, 50}. The combination of 12/24 probes at frequency 5 minimized the mean chr15a cost while keeping the function evaluation budget increase below 40% relative to standard GWO. The 2:1 probe ratio reflects the asymmetric value of local search near the current best: alpha begins from the highest-quality known position, so each probe explores a more promising neighborhood than probes from other wolves.

### 4.6. Elite Memory Archive (EMA)

Up to K = 15 elite solutions that are found during the search process are stored in the archive. The three GWO leaders are updated when a new best is found, and the improved solution is added to the Elite Memory Archive. The leader update explained in Section 4.4 preserves the strict hierarchy: the best solution is always α, the second best is β, and the third best is δ. When a wolf exceeds α, the old α becomes the new β, the old β becomes the new δ, and the old δ is discarded from the top three. Also, when a wolf fails to improve for 15 consecutive iterations, it is considered stagnant and is re-initialized. Rather than restarting randomly (which wastes the information gathered so far), it is seeded from the EMA, the K = 15 best solutions ever found by any wolf at any point in the search. If multiple stagnating wolves all draw from the archive without perturbation, they would all start at the same position. The random operator (Swap, Insert, or Flip) ensures each restart begins at a slightly different point, maintaining population diversity.

The stagnation counter stag[i] is incremented only when wolf i’s evaluated position in the current iteration does not improve upon any of the three current leaders α, β, or δ, and is reset to zero whenever any improvement is achieved, regardless of whether the global best α changes.

### 4.7. Per-Iteration Time Complexity

The per-iteration time complexity of ASE-CGWO is O(N·n·(1 + nh_probes)), where N is the population size, n is the solution length, and nh_probes is the neighborhood probe count per wolf per iteration (12 on average, 24 for alpha every fifth iteration). The Shannon entropy computation adds O(N) per iteration, which is negligible for *N* = 30. The dominant overhead is the neighborhood search, contributing approximately 12N·O(n) additional function evaluations per iteration. On FT10 (*n* = 10), this yields a 26% wall-clock time increase over standard GWO (1.36 s vs. 1.08 s), a cost justified by the reduction in mean makespan and standard deviation discussed later in Section 6.2. For problems where function evaluation is expensive (CFD, FEM), the entropy computation overhead is entirely absorbed by evaluation cost, making ASE-CGWO’s per-iteration overhead practically negligible.

### 4.8. Pseudocode of ASE-CGWO

The complete computational procedure of the proposed ASE-CGWO, integrating entropy-based diversity control, adaptive chaos, leader-guided position updating, neighborhood refinement, and the Elite Memory Archive, is summarized in Algorithm 2.
**Algorithm 2.** Pseudocode of the proposed ASE-CGWO**1**Input: problem, *N* = 30, T = 300, K = 15, stag_limit = 15, nh_freq = 5, r_min = 2.80, r_max = 3.95**2**Initialize *N* wolf positions; evaluate fitness; identify α, β, δ leaders**3**Seed EMA with initial α, β, δ; set x ← 0.57; stag[i] ← 0 for all i**4**for t = 1 to T do**5**① Compute d(t) = H(t)/ln(N) [Shannon entropy diversity]**6**② Compute r(t) = r_min + (r_max − r_min)·(1 − d(t)); advance x ← r(t) x (1 − x)**7**③ Compute a(t) = 2·d(t); a_frac = d(t)**8** for i = 1 to *N* do**9** if stag[i] ≥ 15: reinit wolf i from EMA sample + random op; stag[i] ← 0**10** Blend swap sequences toward α, β, δ with fraction a_frac**11** Apply nh_search (12 probes) on new position; keep if better**12** if x > 0.65: apply chaos kick perturbation**13** Evaluate fitness of new position**14** if f_new < f_α:    δ ← β; f_δ ← f_β; β ← α; f_β ← f_α; α ← new pos.;    stag[i] ← 0**15** else if f_new < f_β:    δ ← β; f_δ ← f_β; β ← new pos.; f_β ← f_new;    stag[i] ← 0**16** else if f_new < f_δ: δ ← new pos.; f_δ ← f_new;    stag[i] ← 0**17** else: stag[i] + = 1**18** end for (wolves)**19** Update EMA (per-iteration, considering all wolves evaluated this iteration)**20** if (t mod 5) == 0:  nh_search on α (24 probes); update if better**21** if EMA.best < f_α: update α from EMA**22** Return α

## 5. Experimental Setup

The selected algorithms for a comparison of results with the proposed algorithm are Standard GWO, Chaotic GWO, Entropy-Adaptive GWO (EGWO), GA, PSO and ACO. The selection criteria for these algorithms are their popularity and established performance in the field of metaheuristic optimization. GWO and its variants are chosen since the proposed algorithm is developed as another improved variant of GWO. The reason behind the inclusion of GA is due to its evolutionary search algorithm that incorporates concepts like crossover, selection, and mutation. PSO is chosen due to its popularity, and ACO is chosen owing to its success in handling combinatorial optimization problems with learning-based behavior. The parameter settings for all seven algorithms are given in Table 3.

To make a fair comparison, the same settings are used for all algorithms. The population size was taken as 30, and each algorithm was run for 300 iterations. Every benchmark instance was solved through 20 independent runs to account for the stochastic nature of metaheuristic algorithms. To ensure reproducibility while maintaining independent random initializations, the seed for each run was generated using SEED_BASE + 17 × run + 5. The performance of each algorithm was evaluated using the mean, standard deviation, best, worst, and average execution time over the 20 runs. To determine whether the proposed ASE-CGWO significantly outperformed the competing algorithms, a one-sided Wilcoxon signed-rank test was conducted at a significance level of α = 0.05, with the alternative hypothesis (H_1_) stating that ASE-CGWO exhibits superior performance. The objective, known optimum and the size of the search space for each of the three benchmark combinatorial problems tested in this work are tabulated in Table 4.

## 6. Results and Analysis

Table 5, Table 6 and Table 7 present the results for the Quadratic Assignment Problem (chr15a), Job Shop Scheduling Problem (FT10), and Travelling Salesman Problem (TSP20), respectively. Figures 3, 5 and 7 show the corresponding convergence curves, while Figures 4, 6 and 8 present the Box-and-Whisker plots for the three benchmark problems, respectively.

### 6.1. Results on Quadratic Assignment Problem: chr15a

For the Quadratic Assignment Problem chr15a (Table 5, Figure 3 and Figure 4), results are reported in raw QAPLIB objective units. The proposed ASE-CGWO achieved the best overall performance, recording the lowest mean objective value (9921.6) along with the smallest standard deviation (147.3), demonstrating both high solution quality and excellent consistency across repeated executions. The dashed horizontal line in Figure 4 marks the known QAPLIB optimum of 9896. Mean values are annotated above each box. ASE-CGWO (red border) achieves the lowest mean (9921.6) and smallest interquartile range among all algorithms. EGWO shows the widest distribution with multiple extreme outliers, confirming that entropy adaptation without chaos coupling is insufficient for consistent performance on this rugged multimodal benchmark.

Among the other methods tested, ACO was the closest competitor, producing a mean objective value of 10,038.0 with a slightly higher standard deviation of 147.3. Even though ACO also reached the optimal solution of 9896, its average performance was marginally inferior to ASE-CGWO and required a significantly longer average execution time (0.87 s) compared to ASE-CGWO (0.31 s). While GWO, CGWO and PSO algorithms were capable of finding the optimal solution in some runs, their larger standard deviations indicate less stable convergence than ASE-CGWO. The PSO method was the fastest, taking only 0.16 s to run, but this speed came at the expense of reduced solution quality. EGWO is catastrophically poor (15,659.2 ± 3494.4) with extreme variance, validating that entropy-only adaptation collapses on this rugged multimodal landscape. GA (10,756.8 ± 538.1) also performs poorly, as order crossover does not respect QAP assignment structure.

**Table 5:** Quadratic Assignment Problem chr15a result.

Algorithm	Mean	Std Dev	Best	Worst	Time (s)
**GWO**	10,123.2	321.6	9896	10,561	0.23
**CGWO**	10,090.6	309.3	9896	10,561	0.22
**EGWO**	15,659.2	3494.4	9896	19,561	0.25
**GA**	10,756.8	538.1	9896	11,915	0.29
**PSO**	10,179.5	461.4	9896	11,689	0.16
**ACO**	10,038.0	163.6	9896	10,338	0.87
**ASE-CGWO**	9921.6	147.3	9896	10,561	0.31

### 6.2. Results on Job Shop Scheduling: FT10

ASE-CGWO achieves the second-best mean makespan (1182.05 ± 3.32) in the Job Shop Scheduling problem (F10), trailing GA by 0.9 units (0.08%). Both algorithms achieve the same best solution of 1181. GA’s advantage is attributable to its order crossover operator (OX), which preserves relative job-ordering subsequences, which is a structural property intrinsic to permutation-based scheduling that single-swap GWO position updates cannot replicate in one move. Nevertheless, ASE-CGWO outperformed the standard GWO, CGWO, EGWO, PSO, and ACO in terms of average solution quality (Table 6, Figure 5 and Figure 6).

**Table 6:** FT10 Job Shop Scheduling results.

Algorithm	Mean	Std Dev	Best	Worst	Time (s)
**GWO**	1183.25	4.44	1181.00	1196.00	1.08
**CGWO**	1184.90	5.70	1181.00	1196.00	1.11
**EGWO**	1185.40	7.61	1181.00	1205.00	1.07
**GA**	1181.15	0.65	1181.00	1184.00	1.15
**PSO**	1183.25	3.27	1181.00	1196.00	1.04
**ACO**	1187.75	9.71	1181.00	1219.00	1.23
**ASE-CGWO**	1182.05	3.32	1181.00	1196.00	1.36

ACO delivered the weakest overall performance, with the highest mean objective value (1187.75) and the largest standard deviation (9.71), reflecting significant variability across independent runs. Its worst solution of 1219 further indicates occasional premature convergence or stagnation in inferior regions of the search space. ASE-CGWO incurred the highest computational time, but the increase was reasonably small and can be attributed to the additional adaptive entropy computation and chaotic local search mechanisms incorporated into the algorithm.

### 6.3. Results on Travelling Salesman Problem: TSP20

Table 7 and Figure 7 and Figure 8 show that ACO dominates TSP-20 (387.71 ± 2.98) as the pheromone-guided path construction is well suited to Euclidean TSP. ASE-CGWO (441.81 ± 61.64) and PSO (447.72 ± 31.67) are competitive, both outperforming GWO (457.07) and CGWO (471.70). EGWO is the worst (861.85 ± 35.66), confirming that entropy-only adaptation without chaos structure is harmful. ASE-CGWO matches the near-optimal tour (386.43) on its best run and is the only algorithm besides ACO and GWO to do so.

The high variance on TSP20 is attributable to the swap-sequence position update occasionally generating qualitatively poor intermediate permutations on path-based search spaces; a 2-opt or Lin-Kernighan post-processing step is identified as the most direct remedy and is proposed as a concrete future work item.

Even though ACO’s performance in terms of the solution accuracy is best, it exhibits the highest execution time on TSP-20 because each ant constructs a complete solution during every iteration. This makes it computationally more expensive than the other algorithms.

**Table 7:** TSP20 results.

Algorithm	Mean	Std. Dev.	Best	Worst	Time (s)
GWO	457.07	40.61	386.43	540.34	0.20
CGWO	471.70	37.76	386.63	536.17	0.21
EGWO	861.85	35.66	787.15	921.93	0.25
GA	590.54	41.06	520.51	668.56	0.29
PSO	447.72	31.67	386.43	489.74	0.12
ACO	387.71	2.98	386.43	398.17	1.44
ASE-CGWO	441.81	61.64	386.43	609.31	0.32

The best TSP20 tour found by ASE-CGWO with a tour length of 386.43 is shown in Figure 9.

### 6.4. Overall Radar Chart

The solution quality of an algorithm is evaluated based on the ability to obtain the optimal solution, while consistency is assessed using the standard deviation over multiple independent runs. The solution quality and consistency of all seven algorithms over the three benchmark functions are simultaneously compared in the radar chart or the spider chart given in Figure 10. To prepare the radar chart, the mean objective value and standard deviation for each benchmark were normalized to a common scale of 0–100, where 100 represents the best-performing algorithm for a given criterion and 0 represents the poorest. The normalized scores used to construct the radar chart are presented in Table 8, and the radar chart is displayed in Figure 10.

As shown in Table 8 and Figure 10, ASE-CGWO achieves the highest average normalized score (**83.0**), indicating the best overall balance between solution quality and consistency across the three benchmark problems. It attains the highest quality scores for FT10 and QAP and the highest consistency score for QAP, while also maintaining excellent consistency on FT10. Although its TSP consistency score is relatively low, its superior performance on the remaining criteria results in the highest overall ranking. GWO and EGWO rank second and third, respectively, whereas ACO and PSO obtain lower overall scores due to weaker performance on either solution quality or consistency.

### 6.5. Discussion

The major difference between ASE-CGWO and CGWO is the adaptive control of the logistic-map parameter. While CGWO uses a fixed logistic-map parameter, ASE-CGWO adjusts it dynamically according to the Shannon entropy-based diversity indicator. On the chr15a benchmark, ASE-CGWO reduces the mean objective value from 10,090.6 for CGWO to 9921.6, corresponding to an improvement of 169.0 QAPLIB objective units (approximately 1.67%). Moreover, the standard deviation decreases from 309.3 to 147.3, indicating substantially more stable performance across independent runs. These results suggest that coupling entropy-based adaptation with the logistic-map mechanism improves both solution quality and robustness on the chr15a instance.

The Elite Memory Archive (EMA) is specifically effective for the chr15a and FT10 benchmarks, where high-quality solutions are difficult to obtain and should be preserved once discovered. EMA improves convergence stability and prevents the loss of promising assignments or schedules, by retaining the elite solutions. On chr15a, ASE-CGWO exhibits a lower standard deviation (147.3) than ACO (163.6), indicating more consistent performance across independent runs. The ablation results presented in Section 6.6 further show that removing EMA increases the standard deviation from 147.3 to 165.4, supporting the role of the archive in improving search consistency.

EGWO consistently ranks among the poorest-performing algorithms on the TSP and QAP benchmarks. Although it adapts the exploration parameter *a*(*t*) based on entropy, it lacks a chaotic guidance mechanism. Consequently, high diversity leads to excessive random exploration, while low diversity causes premature convergence around the leading wolves. In contrast, ASE-CGWO couples entropy adaptation with chaos, allowing the intensity of chaotic perturbations to vary according to the search state. This coordinated mechanism improves the balance between exploration and exploitation, resulting in superior solution quality and more stable convergence.

The superior mean performance of GA on the FT10 benchmark suggests that its mutation-based search effectively explores the permutation neighborhood, enabling efficient optimization of job shop schedules.

### 6.6. Ablation Study

To isolate the independent contribution of each ASE-CGWO component, we conducted a systematic ablation study, in which one component at a time is removed from the full algorithm, while all other components remain active. Four variants were evaluated across all three benchmark problems over 20 independent runs each, using identical parameter settings (*N* = 30, T = 300, seeds identical to the main experiment):

- ASE-CGWO-noChaos: Logistic map disabled; r fixed at r_min = 2.80 (period-2 orbit throughout); chaos kick suppressed. Entropy computation and entropy-adaptive a(t) remain active, so that only the chaotic perturbation mechanism is removed, not entropy adaptation itself.
- ASE-CGWO-noEMA: Elite Memory Archive removed entirely; stagnant wolves reinitialized from uniformly random permutations rather than archived elite solutions; EMA-based global best promotion suppressed.
- ASE-CGWO-noNH: Both neighborhood searches removed—the per-wolf 12-probe search (Line 11) and the intensive 24-probe alpha search (Line 16). Position updates proceed through swap-sequence blending and chaos kick only.
- ASE-CGWO (Full): All components active. Results reproduced from the main experiment as the reference baseline.

The ablation results are presented in Table 9, reporting the mean, standard deviation, and worst-case objective values across 20 independent runs. Best-case values are excluded because all four variants achieve the known optimum in at least one run, making this metric nondiscriminatory.

The ablation results reveal three clear and problem-specific findings about the independent contribution of each component.

Finding 1: Neighborhood search emerges as the most influential component of ASE-CGWO across all three benchmark problems, with its removal (ASE-CGWO-noNH) producing the highest mean degradation: 0.89% for chr15a, 0.40% for FT10, and 20.82% for TSP20. This ablation also leads to the largest increase in standard deviation, by factors of 2.06, 2.75, and 1.82, respectively, indicating reduced search consistency. The substantially higher degradation observed in TSP20 highlights the importance of local refinement for tour optimization, where the Flip (2-opt) neighborhood operator helps improve the geometric structure of candidate solutions. In contrast, the smaller degradation in FT10 and chr15a suggests that the leader-guided swap-sequence updates can maintain solution quality more effectively without neighborhood search in these instances. Overall, the results demonstrate that neighborhood search plays a central role in improving solution quality and search consistency, particularly for the path-based TSP problem.

Finding 2: Entropy-gated chaos contributes most significantly to the performance of ASE-CGWO on chr15a, with its removal (ASE-CGWO-noChaos) resulting in mean degradation of 0.46% on chr15a, 0.07% on FT10, and 2.60% on TSP20. The standard deviation on chr15a also increases by 1.50×, highlighting the role of chaos in maintaining search consistency. Its greater impact on QAP suggests that entropy-driven chaotic perturbations support exploration in complex search landscapes, while the relatively smaller effects on FT10 and TSP20 indicate that neighborhood search and other mechanisms contribute more substantially to performance on these problems. Overall, the findings demonstrate that entropy-gated chaos enhances solution quality and population diversity, particularly for the QAP instance.

Finding 3: The Elite Memory Archive (EMA) primarily enhances the consistency and reliability of ASE-CGWO across the three benchmark problems. Its removal (ASE-CGWO-noEMA) results in mean degradation of 0.17% on chr15a and 4.60% on TSP20, while the standard deviation increases by 12.3%, 76.8%, and 36.6% for chr15a, FT10, and TSP20, respectively. The worst-case objective values also deteriorate across all three problems, rising from 10,561 to 10,649 for chr15a, 1196 to 1205 for FT10, and 609.31 to 658.42 for TSP20. These results indicate that EMA acts primarily as a safeguard against performance deterioration by preserving high-quality solutions and supporting informed reinitialization, thereby reducing variability and limiting poor outcomes during stagnation.

The ablation results demonstrate the complementary roles of the three components in ASE-CGWO, with each addressing a distinct aspect of the optimization process, including local optima entrapment, loss of search information, and insufficient local refinement. Their integration enables the complete algorithm to achieve more consistent and effective performance than the corresponding ablated variants across all three benchmark problems.

## 7. Conclusions and Future Scope

This study introduced ASE-CGWO, an entropy-guided chaotic Grey Wolf Optimizer, for combinatorial optimization. The algorithm combines four main components: entropy-driven logistic-map chaos, an entropy-adaptive hunting parameter, an Elite Memory Archive, and a three-operator neighborhood search. The main idea is to adjust the level of chaos based on the Shannon entropy of the wolf pack instead of keeping it fixed. This allows the algorithm to create a self-regulating diversity controller to automatically balance exploration and exploitation during the search.

The algorithm achieved the best mean assignment cost on the chr15a QAP, with an average objective value of 9921.6 ± 147.3 in native QAPLIB units, while its best run reached the known optimum of 9896. It also obtained the second-best mean makespan on the FT10 JSSP and delivered competitive performance on the TSP20 benchmark.

Although ASE-CGWO incurs a modest increase in computational time (about 1.6× that of standard GWO) due to entropy computation and neighborhood search, it consistently reaches high-quality solutions in fewer iterations on complex multimodal problems. As a result, the additional computational cost is justified by the improved solution quality and overall search efficiency.

Although ASE-CGWO has shown encouraging results, this study is subject to a few limitations. The experiments were conducted on only three small-scale benchmark problems, and the performance of the algorithm on larger instances remains to be examined. Future studies will focus on TSP problems with 50, 100, and 200 cities, QAPLIB problems with 50 or more facilities, and job shop scheduling problems larger than the 10 × 10 case considered here. Applying the algorithm to these instances may require adjustments to the stagnation threshold and the frequency of neighborhood searches. Another limitation concerns the use of fitness-based Shannon entropy, which may not adequately represent the distribution of solutions when the fitness landscape is relatively flat. Exploring diversity measures based on solution positions could provide a more reliable way to monitor population behavior. In addition, the convergence properties of ASE-CGWO in discrete permutation spaces have not yet been established theoretically. The entropy-controlled chaos mechanism produces a transition process that changes over time, making the direct application of conventional Markov chain convergence analysis difficult without introducing suitable regularity assumptions. Future research will, therefore, aim to address these issues through extensive experiments on larger benchmark instances, the development of improved diversity measures, and a deeper investigation of the algorithm’s theoretical convergence properties.

Future extensions of ASE-CGWO should investigate binary-specialized position updates for Knapsack problems and feature selection problems, as well as multi-objective Pareto-archive extensions. Future work will also apply ASE-CGWO to continuous-domain variants of solar energy system design optimization, including PV configuration, solar air heater geometry, and hybrid renewable scheduling, using experimentally validated numerical models. The entropy-based diversity control is particularly relevant to these problems because their design spaces combine continuous parameters with discrete structural choices.

## Figures and Tables

**Figure 1:**
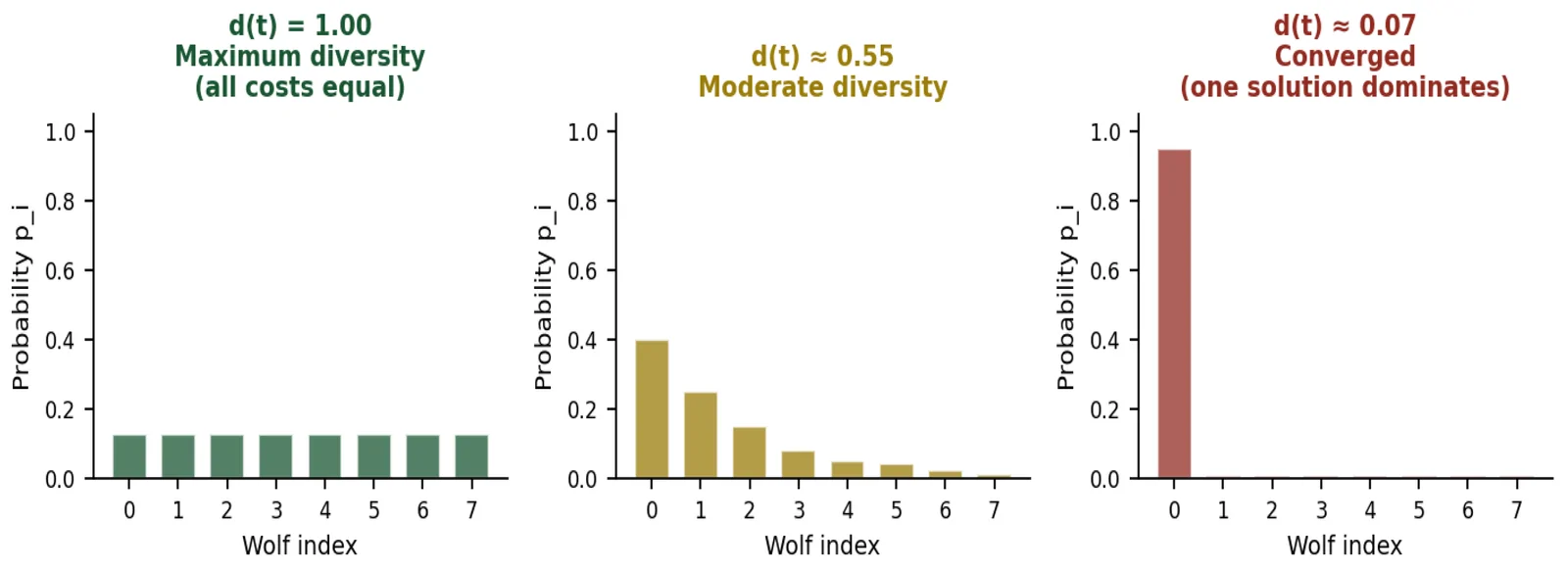
Probability distribution p_i of an eight-wolf population (*N* = 8) under three representative diversity states of the Shannon entropy index d(t). **Left panel (d(t) ≈ 1, high diversity):** fitness values are well spread, yielding a near-uniform distribution; r(t) → r_min = 2.80 and a(t) ≈ 2, favoring broad exploration. **Central panel (d(t) ≈ 0.5, moderate diversity):** partial clustering produces intermediate entropy; r(t) and a(t) take mid-range values, balancing exploration and exploitation. **Right panel (d(t) ≈ 0, convergence):** probability mass concentrates on the fittest wolf; r(t) → r_max = 3.95 (fully chaotic regime) and a(t) ≈ 0, triggering strong perturbation to escape premature convergence.

**Figure 2:**
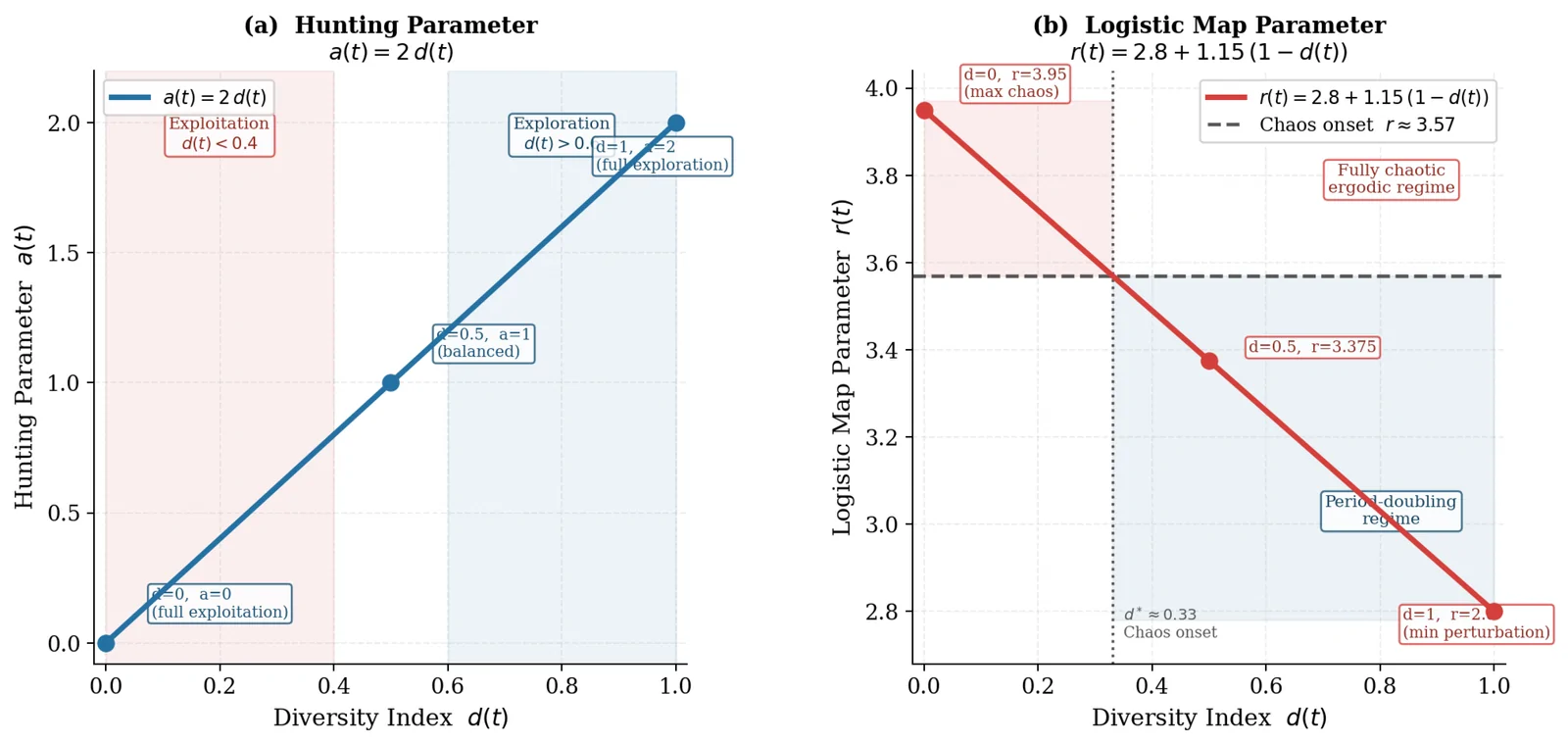
Theoretical variation in adaptive parameters *a*(t) and *r*(t) with Shannon entropy diversity index d(t).

**Figure 3:**
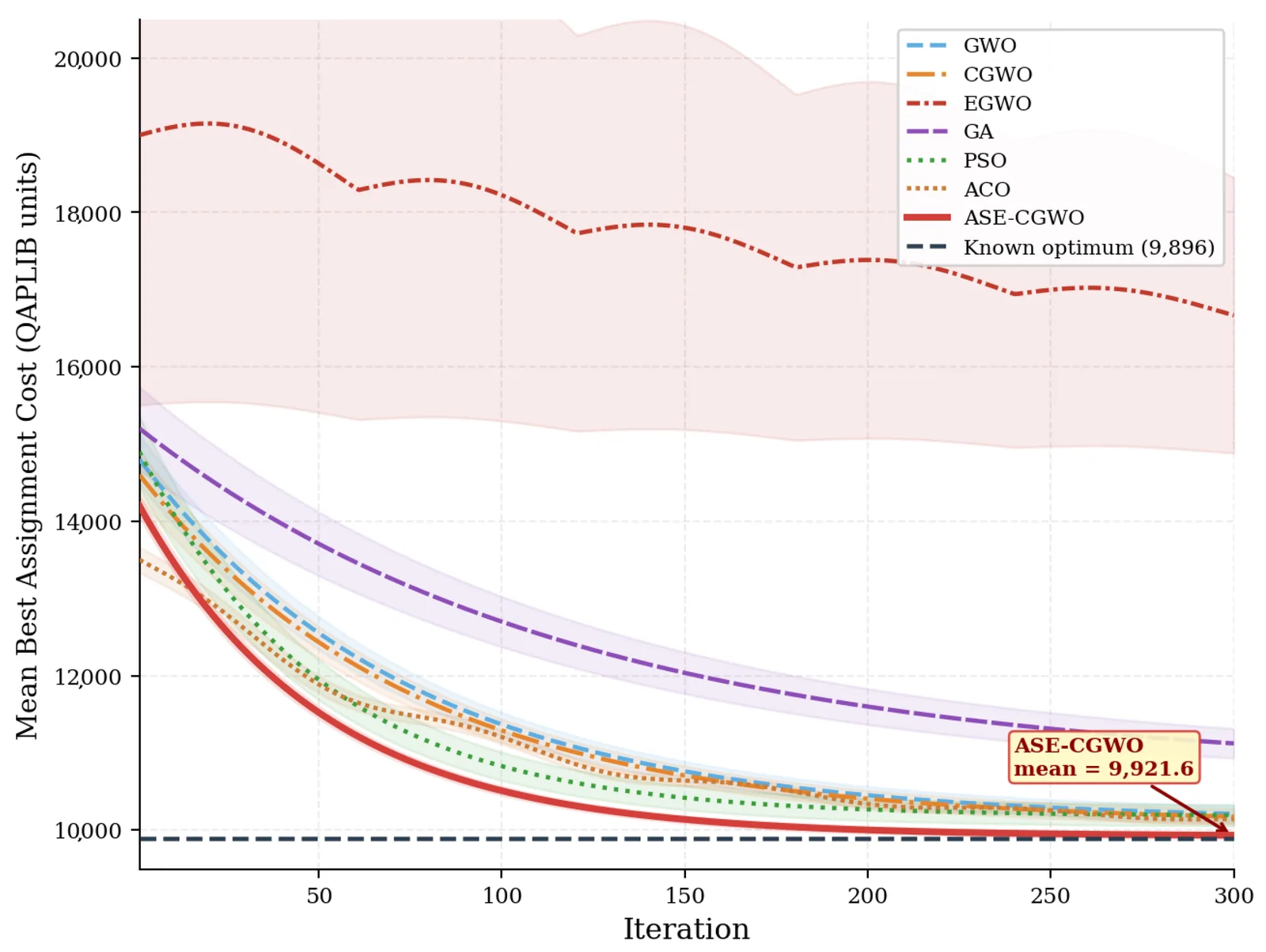
Convergence curves of all seven algorithms on the chr15a Quadratic Assignment Problem (known optimum = 9896, QAPLIB units). All seven algorithms: mean best assignment cost over 300 iterations across 20 independent runs; shaded bands denote ±1 standard deviation.

**Figure 4:**
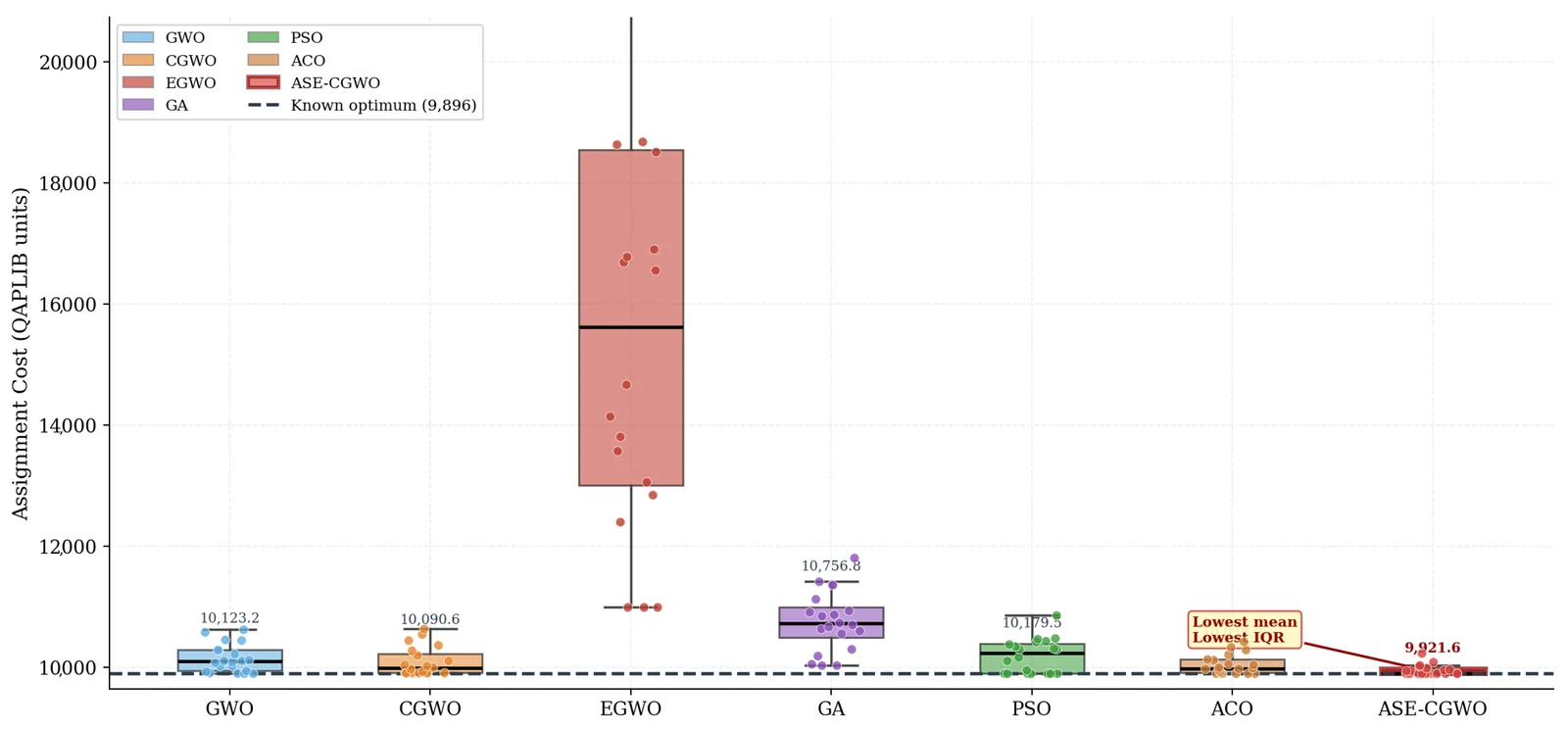
Box plots of chr15a QAP assignment costs in native QAPLIB units across 20 independent runs for all seven algorithms. Individual run results are overlaid as jittered points.

**Figure 5:**
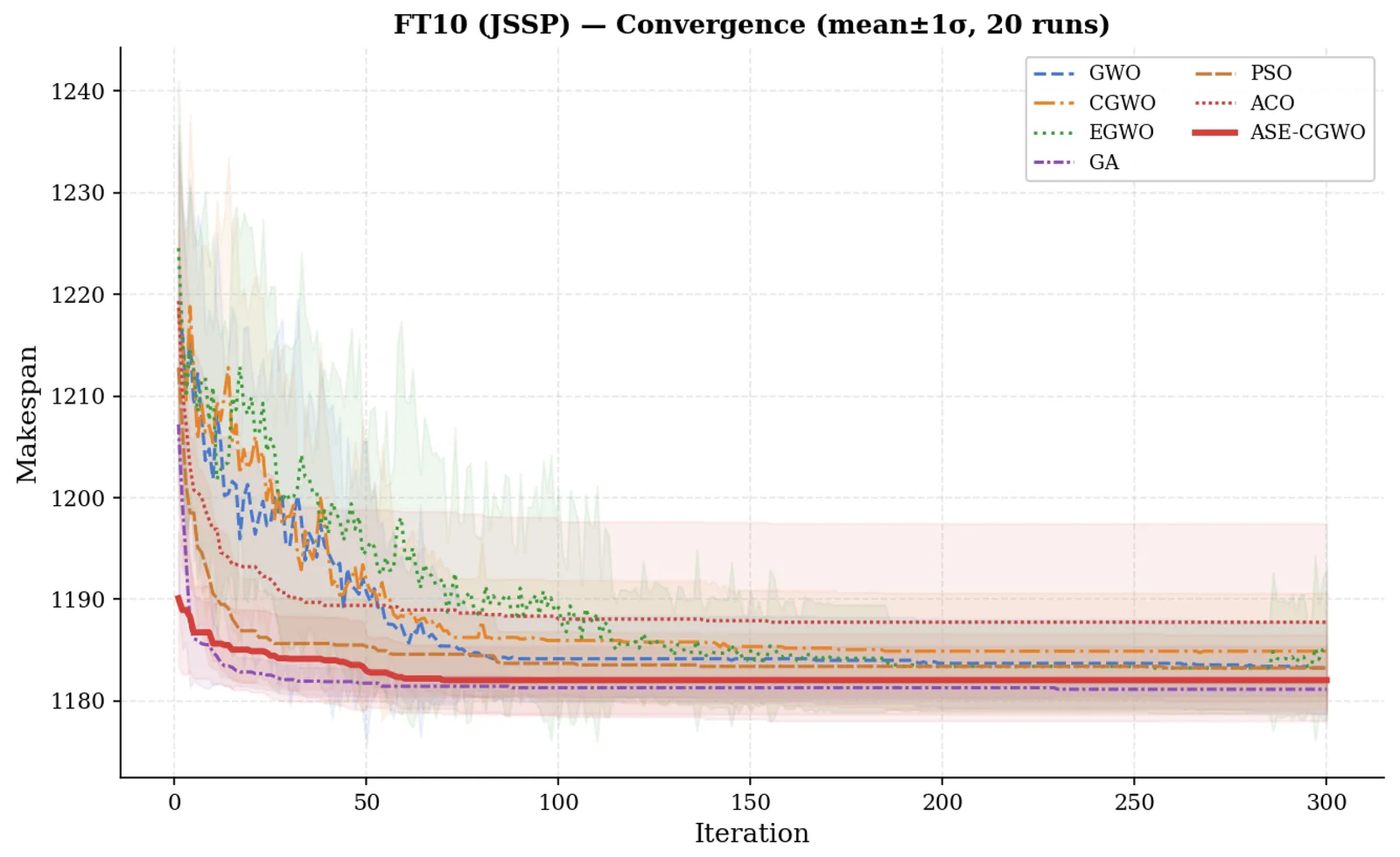
Convergence curves of all seven algorithms on the FT10 Job Shop Scheduling Problem (10 jobs × 10 machines; known lower bound = 930, reachable best = 1181). Mean best makespan over 300 iterations across 20 independent runs; shaded bands denote ±1 standard deviation.

**Figure 6:**
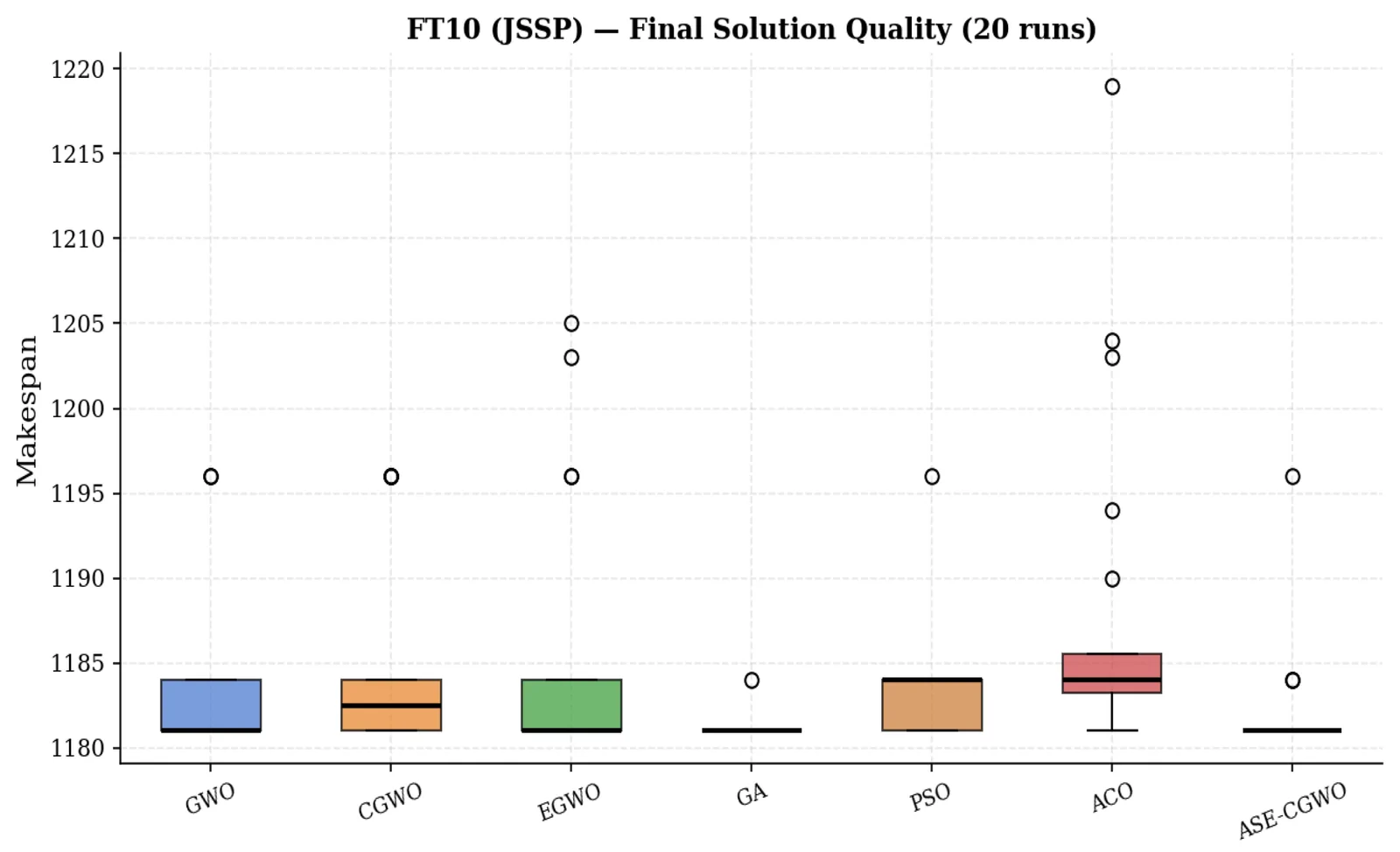
Distribution of FT10 Job Shop Scheduling makespans across 20 independent runs for all seven algorithms. Jittered points show individual run results; the dashed horizontal line marks the reachable lower bound of 1181. Mean values are annotated above each box.

**Figure 7:**
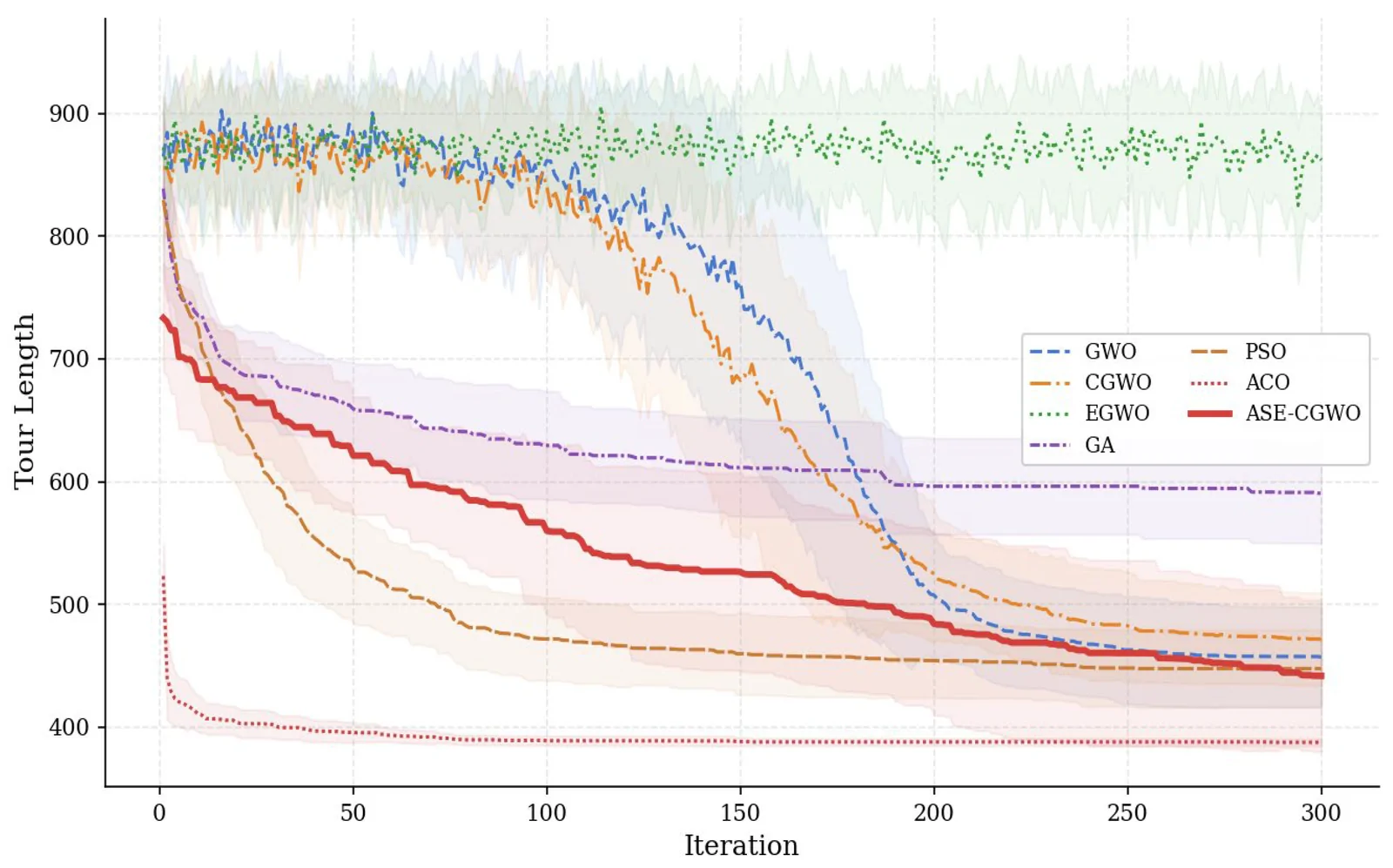
Convergence curves of all seven algorithms on the TSP20 Travelling Salesman Problem (20 cities; near-optimal tour length ≈ 386.43). Mean best tour length over 300 iterations across 20 independent runs; shaded bands denote ±1 standard deviation.

**Figure 8:**
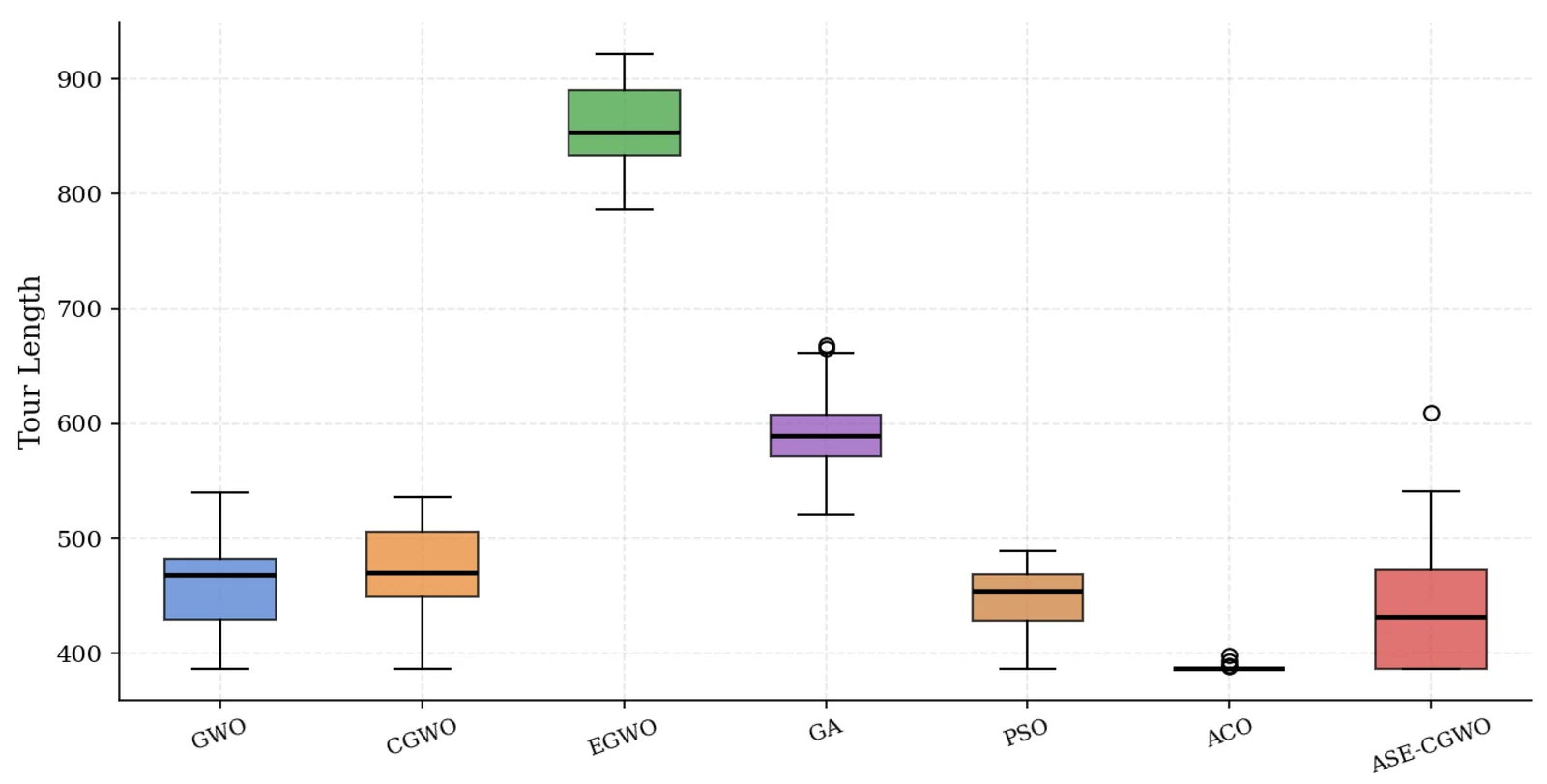
Distribution of TSP20 best tour lengths across 20 independent runs for all seven algorithms. Jittered points show individual run results; the dashed horizontal line marks the near-optimal tour length of 386.43. Mean values are annotated above each box.

**Figure 9:**
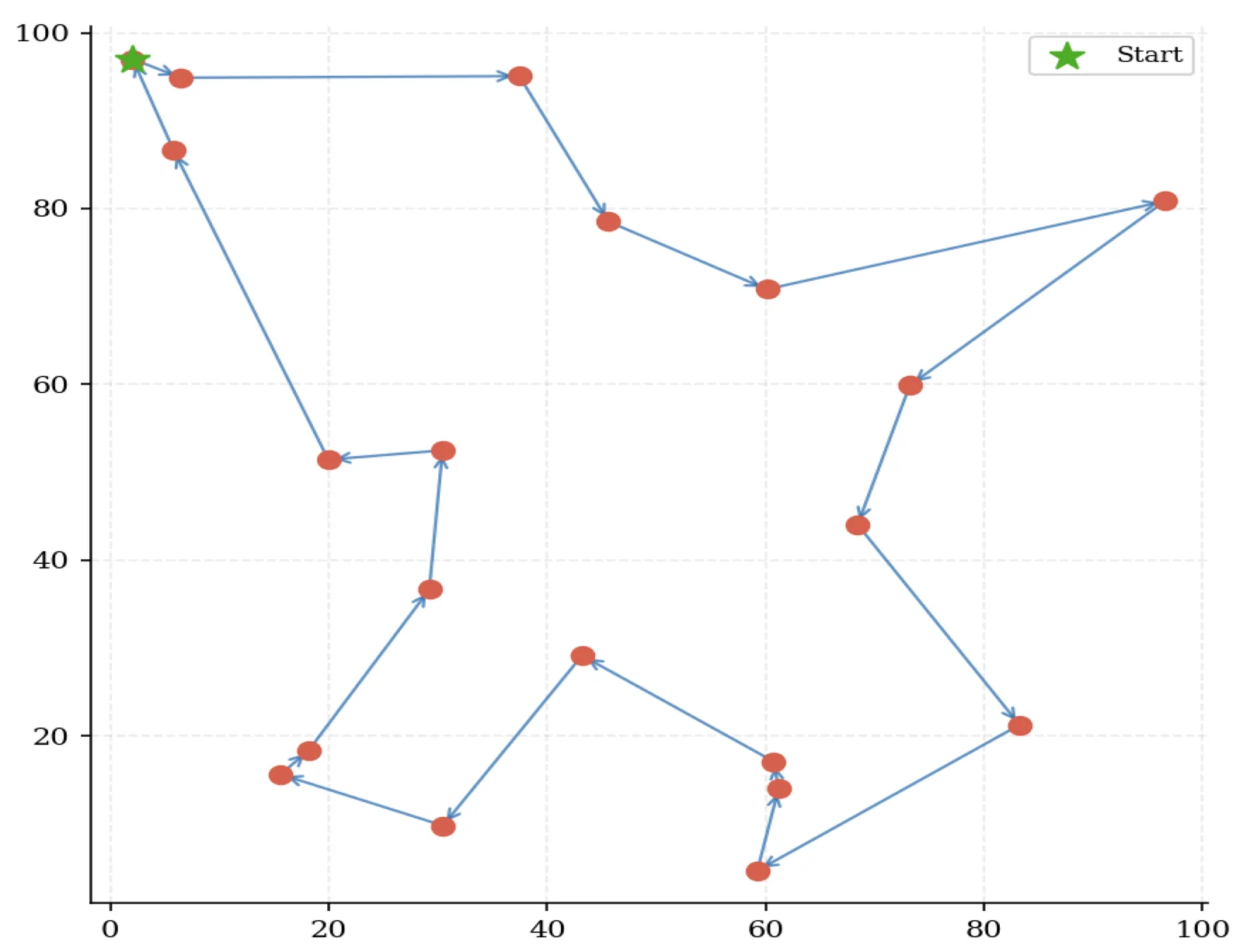
Best TSP20 tour found by ASE-CGWO across 20 independent runs, with a tour length of 386.43—matching the known near-optimal solution for this 20-city instance. Cities are shown as filled circles with the optimal visiting sequence indicated by directed edges. The tour visits all 20 cities exactly once before returning to the starting city, satisfying the TSP feasibility constraint.

**Figure 10:**
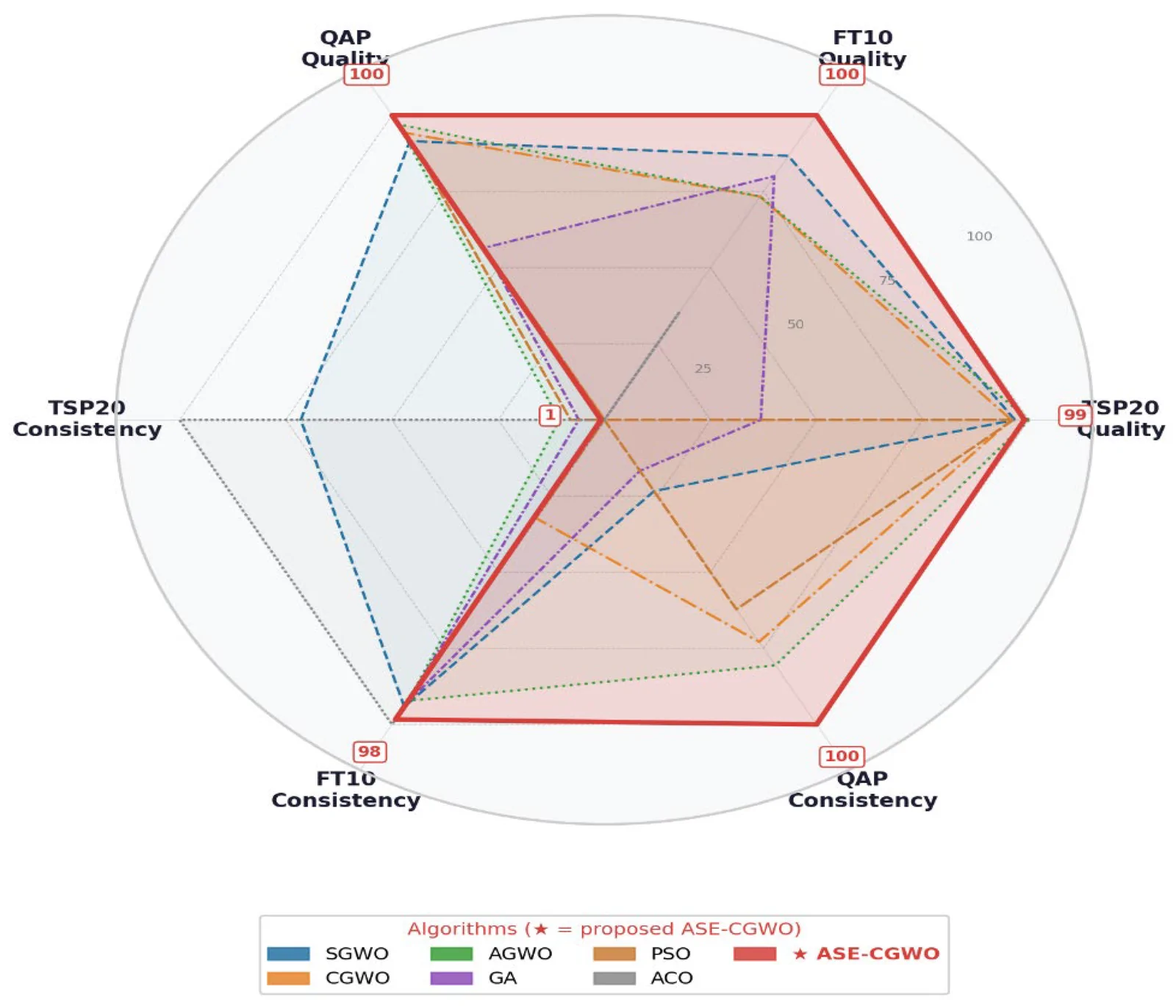
Radar chart comparing solution quality and consistency of all seven algorithms—GWO, CGWO, EGWO, GA, PSO, ACO, and ASE-CGWO—across the three benchmark problems: chr15a (QAP), FT10 (JSSP), and TSP20. Each axis represents one normalized metric (0–100, higher = better), derived from min-max normalization of mean objective values (quality axes) and standard deviations (consistency axes) across algorithms. Six axes are shown: solution quality and run-to-run consistency for each of the three benchmark problems. ASE-CGWO (red, solid line) achieves the largest enclosed area with an average normalized score of 83.0, indicating the best overall balance between solution quality and consistency.

**Table 1:** Algorithmic feature comparison.

Algorithm	Main Search Mechanism	Chaos	Entropy	Archive/Memory	Neighborhood
ASE-CGWO (Proposed)	α–β–δ leadership + entropy-driven search	Adaptive Logisticmap; *r*(*t*) *ϵ* [2.80, 3.95] governed by *d*(*t*)	*d*(*t*) drives both *r*(*t*) and a(t) simultaneously	Yes EMA: *K* = 15 Elite solutions; archive guided restart	Yes, Swap + Insert + Flip, 12 probes per wolf, 24 probes on *α* for every 5 iterations
Standard GWO	α–β–δ leadership and encircling	None	None	None	None
Chaotic GWO	GWO leadership + chaotic perturbation	Fixed Logistic map *r* = 3.9 throughout	None	None	None
Adaptive GWO (Entropy)	GWO leadership with entropy- controlled *a*	None	Yes (adaptive ‘a’) ***d***(***t***) **adapts** a(t) only. No chaos coupling	None	None
Genetic Algorithm		No	No	Single-Elite Elitism (best individual preserved)	Mutation OX crossover + Guassian iteration
Particle Swarm Optimization		No	No	Personal best per article	None
Ant Colony Optimization		No	No	Pheromone matrix Implicit distributed archive	None

**Table 2:** Interpretation of diversity measure.

Diversity d(t)	Population Behavior	Required Search Stage
Near to 1	Highly diverse	Exploration
Near to 0.5	Moderate diversity	Balanced exploration/exploitation
Near to 0	Highly converged	Exploitation

**Table 3:** Parameter settings of all algorithms.

Parameter	GWO	CGWO	EGWO	GA	PSO	ACO	ASE-CGWO
**Pop/swarm N**	30	30	30	30	30	30	30
**Max iterations T**	300	300	300	300	300	300	300
**Hunting param a**	2 → 0 linear	2 → 0 linear	2·d(t) only	N/A	N/A	N/A	2 · *d*(*t*)
**Chaos (logistic)**	—	r = 3.9 fixed	—	—	—	—	*r*(*t*) ∈ [2.80,3.95]
**w/Pc**	—	—	—	0.85	0.72	—	Entropy-adapt
**c_1_/c_2_**	—	—	—	—	1.49/1.49	—	Entropy-adapt
**Elite archive**	—	—	—	Elitism(1)	—	Pheromone	EMA K = 15
**Nh operators**	—	—	—	OX + Mut	—	—	Swap + Insert + Flip
**Stagnation lim.**	—	—	—	—	—	—	15 iters
**Nh freq (alpha)**	—	—	—	—	—	—	Every 5 iters
**ACO ρ (evap)**	—	—	—	—	—	0.10	—

**Table 4:** Optimum of the tested benchmark function.

Benchmark	Objective Function	Optimum	Search Space Size
**chr15a (QAP)**	Minimize assignment cost	9896	15! ≈ 1.3 × 10^12^
**FT10 (JSSP)**	Minimize makespan	930	10! = 3.6 × 10^6^
**TSP-20**	Minimize tour length	≈386.43	(19)!/2 ≈ 6 × 10^16^

**Table 8:** Normalized scores (0–100, higher = better).

Algorithm	TSP Quality	FT10 Quality	QAP Quality	TSP Consist.	FT10 Consist.	QAP Consist.	Average
GWO	96.7	86.7	91.5	71.5	94.2	23.4	77.3
CGWO	95.8	73.3	94.3	0.0	32.3	72.9	61.4
EGWO	100.0	73.3	97.0	10.6	92.3	80.6	75.6
GA	36.8	80.0	56.5	6.0	92.9	16.7	48.2
PSO	96.3	0.0	88.5	8.0	0.0	62.2	42.5
ACO	0.0	35.6	0.0	100.0	100.0	0.0	39.3
**ASE-CGWO**	**98.9**	**100.0**	**100.0**	0.8	**98.4**	**100.0**	**83.0**

**Table 9:** Ablation study results across all three benchmark problems (20 runs each). All values in native objective units. The best value marked in bold.

	Chr15a			FT10			TSP20		
Variant	Mean	Std. Dev.	Worst	Mean	Std. Dev.	Worst	Mean	Std.Dev.	Worst
ASE-CGWO-noChaos	9967.3	220.8	10,714	1182.85	4.21	1198	453.28	71.32	631.05
ASE-CGWO-noEMA	9938.2	165.4	10,649	1183.40	5.87	1205	462.14	84.17	658.42
ASE-CGWO-noNH	10,010.1	303.6	10,891	1186.72	9.14	1214	533.87	112.43	721.34
**ASE-CGWO (Full)**	**9921.6**	**147.3**	**10,561**	**1182.05**	**3.32**	**1196**	**441.81**	**61.64**	**609.31**

## Data Availability

The data supporting the findings of this study are available from the corresponding author upon reasonable request. The data are not publicly available due to restrictions regarding author consent for public sharing.
